# Prevalence of obesity and its association with fast-food consumption and physical activity: A cross-sectional study and review of medical students' obesity rate

**DOI:** 10.1016/j.amsu.2022.104007

**Published:** 2022-06-18

**Authors:** Moustafa Alhashemi, Wafik Mayo, Mohammed Moutaz Alshaghel, Muhamad Zakaria Brimo Alsaman, Luma Haj Kassem

**Affiliations:** a Faculty of Medicine, University of Aleppo, Aleppo, Syria; b Mashabek, Aleppo, Syria; c CME Office, Faculty of Medicine, University of Aleppo, Aleppo, Syria; d MD, ENT Department, University of Aleppo, Faculty of Medicine, Aleppo, Syria

**Keywords:** Aleppo University, Cross-sectional study, Dietary habits, Fast-food consumption, Medical students, Obesity, Overweight, Physical activity

## Abstract

**Background:**

obesity is a widespread condition with more than 400 million obese-person all over the world. It is expected of medical students to follow healthy habits as they are more familiar with the risks of obesity. In contrast, many studies showed that medical students eat fast food, spend much time on smart devices, and do not play sports.

**Aim:**

This study is the first cross-sectional study conducted to evaluate the prevalence of obesity and some daily habits carried out by medical students of Aleppo University.

**Method:**

The data of our cross-sectional study were collected between the 13th-20th of March 2021 through an online google form posted on different social media platforms. The questionnaire was divided into two main sections: firstly, participants' demographics (including age, gender, and academic year). In addition, medical students were asked to document their weight and height to estimate their Body Mass Index (BMI). The second section contained questions concerning daily habits including, the number of meals, breakfast, physical exercises, and fast food consumption. Chi-square and Fisher tests were performed by IBM SPSS statistics to analyze our data.

**Results:**

Our sample size was 514 medical students of Aleppo University. (22.1%) of them were overweight or obese (BMI> 25). Most (73.5%) eat fast food once a week or more. In addition, (39%) have three meals or more daily, and (55.7%) have two meals every day. Regarding physical activity, (76.7%) do not play sports, and (62.1%) use buses to come to the college.

Stress eaters were associated significantly(p-value<0.05) with being overweight, and (70.8%) of them had a history of being overweight (p-value<0.05). Contrary to females, we found that most males were not stressed-eaters (p-value<0.05).

## Highlights

• It is the first cross-sectional study that evaluated obesity prevalence and daily habits by students at Aleppo University.
• The prevalence of obesity according to WHO category was (22.1%). Most students eat fast-food once a week at least (73.5%).
• More awareness campaigns are recommended to raise attention.
• More epidemic studies should be conducted to assess all predisposing factors in this population.

## 1 Background

Obesity is characterized by excessive adipose tissue accumulating in the body; it is classed as a risk factor for various diseases, especially cardiovascular diseases [1]. However, there are no evident underlying causes for obesity, and its mechanisms are unknown. Nevertheless, it results from hereditary, financial, and social impact factors. Moreover, food habits, home environment, lifestyle, sleeping times, and daily activities influence the spread of this condition [2].

During the past thirty years, the obesity rate has increased to 27.5% for adults and 47.1% for youths. Due to its high spread throughout recent years, it is now seen as a pandemic with more than 400 million obese people over all the world [2]. BMI is used to estimate obesity and overweight rates where BMI of 30 kg/m2 or higher is considered obese [1]. Whitlock G et al. found that every 5-degree increase in BMI causes a higher mortality rate by 29%, 210% for mortalities because of diabetic Mellitus, and 41% for vascular diseases [3].

Fast food is pre-packaged food that is usually related to high calories. There is no precise and agreed definition of fast food in the field of scientific research [4]. However, fast-food consumption, the massive portion size of the meals, and increased amounts of sugar are undeniably related to overweight and obesity [1].

University students were exposed to unhealthy habits for many motivations during their study years, increasing their risk of physical problems [5,6]. On the contrary, medical students are expected to adopt a healthier lifestyle considering their advanced scientific knowledge of food compounds and their effect on the body. Regardless, doctors advise people to follow healthy habits as they do not follow them themselves [7]. Following unhealthy diets by healthcare providers might influence their work performance. However, this is the first study that assesses the prevalence of obesity and fast food consumption in medical students of Aleppo University in Syria and shows the association between obesity and some daily habits.

## 2 Methods

### 2.1 Study design, setting, participants, and data collection

This is a cross-sectional study conducted to assess the prevalence of obesity, dietary habits, and physical activities among medical students at Aleppo University. The targeted population was all medical students from all years of nearly 6000 students.

The study population included 514 medical students of Aleppo university from the first to the sixth year. Pregnant students and any responses from outside the college were excluded or those who did not answer the weight or height.

A previous questionnaire developed by Asghar A et al. [8], to calculate obesity and assess dietary and physical daily habits for medical students of Karachi was used in our study. The questionnaire was created using Google Form, then published online on the college's social media platforms-mainly on Facebook and Telegram groups-each year from first to sixth. Data was collected between the period of 13th-20th of March 2021. The questionnaire was in English using simple terminology, uncomplicated to understand by all participants.

Our work is fully compliant with the STROCSS 2021 criteria [32].

### 2.2 Sample size

EpiInfo-software v7.2.4.0, provided by The Centers for Disease Control and Prevention (CDC), was used to calculate the sample size, 361 participants.

### 2.3 The survey

The questionnaire consisted of two main sections. The first one was for the socio-demographic characteristics of the participants, including their age, gender, and academic year. The number of the student's ID was optional to mention, and it was requested to document the forms and avoid repetition. In addition, bodyweight (in kilograms) and height (in meters) were also recorded to calculate BMI for all participants.

The second section was on nutritional habits and exercise routine. It focused on the type and quality of food, the frequency of eating fast food, having breakfast in the diet, consuming juices, sugary materials, chocolate, etc. As for physical activity, questions on playing any sport or exercise were asked.

### 2.4 Ethics approval and registration

Ethical approval has been granted by the Institutional review board (IRB) of the Faculty of Medicine at Aleppo University (reference number 1535) that was compatible with the Declaration of Helsinki. Informed verbal consent was obtained from all participants which was compatible with our institutional approval.

We registered our study in ResearchRegistry in accordance with the Declaration of Helsinki. Our number registry is researchregistry7939 (https://www.researchregistry.com/browse-the-registry#home/registrationdetails/628a27ad01b732001e2f81e7).

### 2.5 Statistical analysis

Data was exported to Microsoft Excel 365 version 10. Statistical analysis was performed using Statistical Package for Social Sciences (SPSS) version 25. Results of the various questions were presented as frequencies and percentages. The results of BMI for all included participants were classified based on the WHO standard classification [9]. When BMI is < 18.5, considered underweight, where the average weight is 18.5–24.9. Overweight is 25–29.9, Obesity class 1 is for BMI between 30 and 34.5, Obesity class 2 is 35–39.9, and obesity class 3 is for BMI above 40. Means with standard division were also calculated for every category of BMI.

From 1st to 3rd year, academic years are considered preclinical years, while the other years are regarded as clinical years at the University of Aleppo. Therefore, for inferential analysis, the participants’ academic years were merged into two categories, preclinical and clinical academic years. The BMI was calculated to evaluate the effect of dietary habits and physical activity on weight statutes. According to the WHO classification of BMI, groups from 3 to 6 were grouped as one category, “Overweight".

Chi-square and Fisher's exact tests were used for categorical data, and the threshold statistical significance was determined by alpha value equals 0.05.

## 3 Results

### 3.1 Demographic characteristics

A total of 514 medical students from all the years respond to our survey, where 276 (53.7%) were females and 238 (46.3%), were male. The third-year students represented the sample's highest percentage (25.3%), while only 51 (9.9%) participants were in the first year; 53% were between 21 and 23 years-old. In addition, the majority of our sample 501 (97.5%) were single. Table (1).

**Table 1:** General characteristics.

Characteristics (total n = 514)	n (%)
**Gender**	
Male	238 (46.3)
Female	276 (53.7)
**Year of study**	
1st year	51 (9.9)
2 nd year	87 (17.0)
3rd year	130 (25.3)
4th year	103 (20.0)
5th year	75 (14.6)
6th year	68 (13.2)
**Age**	
18-20	207 (40.3)
21-23	272 (52.9)
24-27	35 (6.8)
**Marital status**	
Single	501 (97.5)
Married	7 (1.3)
Divorced	3 (0.6)
Widowed	3 (0.6)

BMI classification according to WHO showed that 59 (11.5%) of our sample were underweight, 342 (66.5%) had an average BMI with mean and standard deviation (21.68 ± 1.78), and the rest were in a pre-obesity group 82 (16.0%) or an obesity group 31(6.1%) with BMI more than 30. Table (2).

**Table 2:** Body mass Index classification (Total = 514).

	**N (%)**	**Mean ± Std**
Group1(Underweight): BMI<18.5	59 (11.5)	17.21 ± 1.15
Group2(Normal weight): 18.5≤BMI<25	342 (66.5)	21.68 ± 1.78
Group3(Pre-obese): 25≤BMI<30	82 (16.0)	27.01 ± 1.41
Group4(Obese class1): 30≤BMI<35	24 (4.7)	32.09 ± 1.46
Group5(Obese class2): 35≤BMI<40	6 (1.2)	37.58 ± 1.63
Group6(Obese class3): 40<BMI	1 (0.2)	43.94

### 3.2 Dietary and physical habits

Despite that most of the participants, 457 (88.9%) were aware of the health risk related to fast food, only 27% reported not eating fast food. On the other hand, 378 (73.5%) eat fast food once or more every week. More than half (55.7%) of the participants have two meals every day, 201 (39%) have three meals or more daily, and 5.3% have only one male a day. Nearly 48% of the participants have breakfast regularly, and 77 (15.0%) reported they rarely have breakfast. In addition, 146 (28.4%) of our sample eat snacks daily, 43% percent of the participants described themselves as stress eaters. However, 50% of the participants reported checking their weight regularly.

About the physical activity, 180 (35.0%) of the participants do not exercise, and 394 (76.7%) do not play sports. On the other hand, 120 (23.3%) play different types of sports. Twenty-four percent practice “swimming, running or other types of sports”. Nearly 319 (62.1%) of the students in this study use the bus as the main transport to the college, and only a few use cars (9.7%). Meanwhile, during their free time, reading books was the most practiced activity with 173 (33.7%), while playing sports was a minor favourable activity 26 (5.0%) Table (3). Alternatively, surfing the internet and watching videos were exceptionally favourable activities for nearly 29% of each participant. Almost 45% of the participants spend more than 3 h on the internet. More than half of the participants, 270 (52.5%), have a family history of obesity where at least one family member is overweight.

**Table 3:** Dietary and physical habits (total = 514).

	n (%)		n (%)
How much fast food do you consume in a week?		Family overweight?	
None	136 (26.5)	Yes	270 (52.5)
Once a week	228 (44.4)	No	244 (47.5)
Twice a week	91 (17.7)	Method of transport?	
Thrice a week	29 (5.6)	By foot	145 (28.2)
More	30 (5.8)	By car	50 (9.7)
Are you aware of the risk factors of fast food/soft drink consumption?		By Bus	319 (62.1)
Yes	457 (88.9)	Checking weight regularly?	
No	57 (11.1)	Yes	262 (51.0)
How many meals do you take in a day?		No	252 (49.0)
1 meal	27 (5.3)	How much time do you spend on social media/internet daily?	
2 meals	286 (55.7)	1 h	42 (8.2)
3 meals	158 (30.7)	2 h	104 (20.2)
4 meals	30 (5.8)	3 h	139 (27.0)
More	13 (2.5)	More than 3 h	229 (44.6)
How often do you eat breakfast?		Do you play sports?	
Every day	246 (47.9)	No	394 (76.7)
Alternate day	57 (11.1)	Yes	120 (23.3)
Sometimes	134 (26.0)	Football/basketball	67 (55.8)*
Rarely	77 (15.0)	gym’ or playing cardio”	29 (24.2)*
Are you a stress eater?		Swimming/running/other types of sports	24 (20)*
Yes	175 (34.0)	Do you perform exercise?	
No	339 (66.0)	No	180 (35.0)
How frequently do you consume snacks?		Yes	189 (36.8)
Every day	146 (28.4)	Maybe	145 (28.2)
Alternate day	172 (33.5)	What do you do in your spare time	
Sometimes	112 (21.8)	Playing sports	26 (5.0)
Rarely	84 (16.3)	Watching movies	151 (29.4)
How often do you consume soft drinks/juices/energy drinks?		Reading books	173 (33.7)
Every day	62 (12.1)	Internet and social media	153 (29.8)
Alternate day	32 (6.2)	Other	11(2.1)
Sometimes	172 (33.5)		
Rarely	248 (48.2)		
How frequently do you consume red meat?			
None	138 (26.9)		
Once a week	169 (32.9)		
Twice a week	108 (21.0)		
Thrice a week	68 (13.2)		
More	31 (6.0)

**Table 4:** Associations between characteristics and gender, years of study and Body mass Index classification.

Variables	BMI groups			P-value	Gender		P-value	Years of study		P-value
	Underweight (BMI <18.5) N = 59	Normal (BMI = 18.5–24.9) N = 342	Overweight or obese (BMI >25) N = 113		Female N = 276	Male N = 238		Pre-clinical N = 268	Clinical N = 246	
Are you a stress eater?				0.001†‡			0.000*‡			0.710*
No	41 (69.5)	240 (70.2)	58 (51.3)		160 (58.0)	179 (75.2)		179 (66.8)	160 (65.0)	
Yes	18 (30.5)	102 (29.8)	55 (48.7)		116 (42.0)	59 (24.8)		89 (33.2)	86 (35.0)	
Are you aware of the risk factors of fast food/soft drink consumption?				0.051†			0.207*			0.000*‡
No	12 (20.3)	35 (10.2)	10 (8.8)		26 (9.4)	31 (13.0)		45(16.8)	12 (4.9)	
Yes	47 (79.7)	307 (89.8)	103 (91.2)		250 (90.6)	207 (87.0)		223 (83.2)	234 (95.1)	
Family overweight				0.000†‡			0.217*			0.093*
No	37 (62.7)	174 (50.9)	33 (29.2)		124 (44.9)	120 (50.4)		137 (51.1)	107 (43.5)	
Yes	22 (37.3)	168 (49.1)	80 (70.8)		152 (55.1)	118 (49.6)		131 (48.9)	139 (56.5)	
Checking weight regularly				0.498†			0.479*			0.064*
No	33 (55.9)	163 (47.7)	56 (49.6)		131 (47.5)	121 (50.8)		142 (53.0)	110 (44.7)	
Yes	26 (44.1)	179 (52.3)	57 (50.4)		145 (52.5)	117 (49.2)		126 (47.0)	136 (55.3)	
Do you play sports?				0.253†			0.000*‡			0.531*
No	49 (83.1)	255 (74.6)	90 (79.6)		242 (87.7)	152 (63.9)		202 (75.4)	192 (78.0)	
Yes	10 (16.9)	87 (25.4)	23 (20.4)		34 (12.3)	86 (36.1)		66 (24.6)	54 (22.0)	
Do you perform exercise?				0.914†			0.012*‡			0.040*‡
No	23 (39.0)	121 (35.4)	36 (31.9)		90 (32.6)	90 (37.8)		96 (35.8)	84 (34.1)	
Yes	20 (33.9)	126 (36.8)	43 (38.0)		93 (33.7)	96 (40.3)		86 (32.1)	103 (41.9)	
Maybe	16 (27.1)	95 (27.8)	34 (30.1)		93 (33.7)	52 (21.9)		86 (32.1)	59 (24.0)	
What do you do in your spare time				0.273†			0.000*‡			0.134†
Playing sports	2 (3.4)	17 (5.0)	7 (6.2)		4 (1.5)	22 (9.3)		17 (6.3)	9 (3.7)	
Watching movies	13 (22.0)	96 (28.0)	42 (37.2)		76 (27.5)	75 (31.5)		72 (26.9)	79 (32.1)	
Reading books	21 (35.6)	116 (33.9)	36 (31.9)		112 (40.6)	61 (25.6)		83 (31.0)	90 (36.6)	
Internet and social media	22 (37.3)	107 (31.3)	24 (21.2)		79 (28.6)	74 (31.1)		89 (33.2)	64 (26.0)	
other	1 (1.7)	6 (1.8)	4 (3.5)		5 (1.8)	6 (2.5)		7 (2.6)	4 (1.6)	
Method of transport				0.076†			0.106†			0.239†
By foot	14 (23.7)	103 (30.1)	28 (24.8)		69 (25.0)	76 (31.9)		81 (30.2)	64 (26.0)	
By car	2 (3.4)	31 (9.1)	17 (15.0)		32 (11.6)	18 (7.6)		21 (7.8)	29 (11.8)	
By Bus	43 (72.9)	208 (60.8)	68 (60.2)		175 (63.4)	144 (60.5)		166 (62.0)	153 (62.2)	

### 3.3 Associations between characteristics and gender, years of study, and Body mass index classification

The overweight participant's group had the highest rate of stress eating habits (48.7%); they also had a significantly higher rate of family history of being overweight (70.8%) with a statistically significant association (P-value<0.05) There was no significant difference between BMI groups concerning awareness of fast food or soft drink consumption risk factors. The underweight group was the least aware of those risk factors (79.7%). There was no significant association between BMI group and checking weight regularly, performing an exercise, or playing sports. The participants with average weight reported playing sport the most (25.4%). All participants did not report “playing sport” as the main activity in their spare time. They preferred reading books in similar proportions. The participants in the underweight group used buses more than the other groups to come to their college 72.9%, while participants with normal range BMI were the most to walk to their college 30.1% (p-value<0.05), However, they checked their weight regularly more than other participants.

**Fig. 1:**
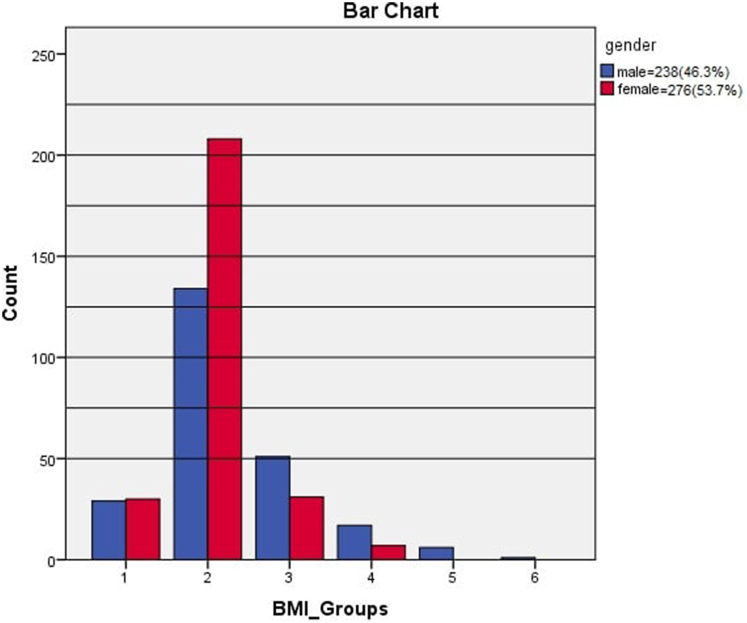
BMI groups according to the Gender.

About the gender, most males did not follow stress eating habits (p-value<0.05), contrary to females (p-value<0.05). Clear dominance was found for the male gender in pre-obesity and obesity groups, while the female gender was more prominent in a normal group in (Fig. 1). Males reported playing sports and performing exercises more than females (p-value<0.05). There was no difference between males and females checking their weight regularly and using transportations. Both genders use the bus as the primary method to get to their college (more than 60% for each). Most females preferred reading books in their free time, while males preferred watching movies.

Participants in the clinical years of study were more aware of the risk factors of fast food consumption. As a result, they performed exercises more than their colleagues in the preclinical years (p-value< 0.05, p-value = 0.040, respectively). However, 62% of participants in clinical or preclinical years used the bus to get to their college. Thirty-six percent of students in clinical years spend their free time reading books, while their colleagues (33.2%) in preclinical years spend their time on the internet.

## 4 Discussion

Excess body weight during a lifetime is associated with an increased risk of many diseases and early mortality rates [10]. While medical students are considered reasonably informed concerning health issues and adopt healthy practices, they consume alarming fast-food levels. In this investigation, we conducted a cross-sectional study in which we assessed BMI status in medical students and their correlation with fast food consumption and dietary practices and healthy attitudes (physical activity behaviour, family factor, social media, and others). To the knowledge of the authors, there is no published study regarding the prevalence of obesity in medical students in Aleppo, especially during the Syrian crisis. All current data is from countries in different geographic regions.

In this study, the prevalence of obesity in our sample is close to other medical students in Malaysia, India, Saudi Arabia, and Sudan whereas Bahrain and Pakistan medical students have the highest rate of obesity. In general, there is a higher incidence of obesity among males than females (Table 5).

**Table 5:** The prevalence of obesity among medical students in different countries.

author	year	country	design	sample size	age (range)	male %	female %	prevalence of obesity (BMI> 30)		
								total	male	female
alhashemi et al.	2021	Syria	CS study	514	18–27	46.30%	53.70%	6.10%	10.08%	0.02%
Gopalakrishnan et al. [23]	2012	Malaysia	CS study	290	19–25	45.20%	54.80%	5.20%	9.20%	1.90%
Purohit et al. [24]	2015	India	CS study	138	18–25	NM	NM	13.04%	NM	NM
Soma et al. [16]	2009	Bengal	CS study	114	NM	61.40%	38.60%	3.40%	3.40%	0%
Mahmood et al. [25]	2013	Pakistan	CS study	428	18.5–21.9	35.30%	64.70%	12.40%	47.70%	15.90%
Asghar et al. [26]	2019	Pakistan	CS study	351	17–25	26.50%	73.50%	18.40%	27.90%	15.10%
Mehmood et al. [27]	2016	Saudi Arabia	CS study	405	19–25	41.70%	58.30%	8.40%	NM	NM
Chourdakis et al. [28]	2010	Greece	CS study	390	19.6–23.4	47.95%	52.05%	3.50%	5.90%	1.50%
Bede et al. [29]	2020	Cameroon	CS study	203	17–27	44.30%	55.70%	3%	1.10%	3.50%
Yousif et al. [30]	2019	Sudan	CS study	216	18–25	42%	58%	6.50%	NM	NM
Ahmed et al. [31]	2019	Bahrain	CS study	251	17–25	32.70%	67.30%	16.00%	15.90%	16.10%
Shah et al. [1]	2014	India	CS study	147	NM	NM	NM	9.52%	NM	NM

CS: Cross Sectional, NM: Not Mentioned.

In this study, medical students' fast food consumption based on the weekly food consumption assessment cannot be denied as 29.1% of students are taking two meals or more every week. Long college days, combined with daily fatigue, can lead to fast food being a popular choice for medical students. Moreover, most fast-food restaurants in Syria do not display calories in their meals or sandwiches. This results in increased people's consumption of fast food, particularly medical students [11]. In addition, there was a high prevalence of snack consumption as 33.5% and 28.4% used to eat snacks daily or alternate days. Additionally, more than half of our sample (52.1%) used to skip their breakfast, which could be related to increased consumption of fast food, snacks, and soft drinks.

Most of the students (88.9%) were aware of the risk factors of fast food/soft drinks. This could be associated with more than half of the sample (52.5%) having a family history of being overweight or obese, as the relationship between social role or genetics and the possibility of an individual being obese [12]. The results of our study revealed that most of the sample had varying numbers of meals per day with two main meals as a majority, which was similar to other studies [13].

This study distinguished one aspect of eating behaviours, as more than a third of the sample (34%) were stress eaters. With some crucial associations, females were twice as many as males. Half of them were overweight or obese, as well as half of them had a family history of being overweight or obese. This was consistent with a previous study that revealed a significant relationship between emotional eating and BMI [14].

Physical activity or playing sports are essential factors in maintaining health, significantly reducing chronic disease risk, preventing excessive weight gain, and improving physical fitness [15]. However, our study found that 76.7% of the sample do not play sports. This significantly low level of physical activity could be attributed to medical students' lack of time and study pressure and a small number of places available for various types of sports. Moreover, our study revealed a negative relationship between physical activity level and BMI. This finding is consistent with similar studies, which revealed a negative association between physical activity level and prevalence of obesity [16,17]. Nevertheless, other studies showed either a positive relationship [18,19] or no significant association [[20], [21], [22]].

Another important finding was that higher students' academic level enhanced their theoretical knowledge regarding fast food/soft drink risk factors. As unawareness of the risk factors was higher in preclinical students, the percentage was triple compared to those in the clinical stages. (16.8% vs. 4.9%).

A significant advantage of conducting the data through an online questionnaire is participants were able to share their information in a truthful manner, as some people would be embarrassed to tell their actual weight or their unhealthy habits; this meant the information collected is reliable and not biased; this allowed authors to precisely estimate the prevalence of overweight and obesity in our study. Moreover, males and females had similar results that can be used to compare the fundamental differences in obesity, fast food consumption, and physical activity between males and females. On the other hand, there are certain limitations of the study. First of all, our study's data on dietary knowledge, behaviour, and physical activity relied on students' self-reported accounts; therefore, it could have accuracy problems. However, because students understood that their data would be kept confidential, they gave correct responses regarding their behaviour and knowledge. Secondly, the relationship between BMI and fast food consumption at Aleppo University has not been studied in detail before. Therefore, we could not compare our results with previous studies on medical or other college students.

However, medical students are believed to be a significant proportion of the future's socio-economic elite, so their behaviours are most likely to become the norm [22]. Therefore, future policies should increase and encourage awareness and healthy habits. Furthermore, it is necessary to reduce obesity rates among youth, as it is associated with several metabolic and cardiovascular complications in the future like the general populations of their countries.

Further research is needed to understand fast food's contribution to obesity problems and how it has changed after the Syrian crisis, the turmoil of the economic situation, and the psychological harms that accompanied it.

## 5 Conclusion

Over time, the prevalence of overweight and obesity is increasing worldwide. Medical students in Aleppo are consuming fast food and practicing unhealthy habits and obesity is a common phenomenon among them. Although more awareness campaigns are recommended to raise attention, more scientific studies should be conducted to research these behaviours' motivations and complications.

## Ethics approval and consent to participate

All participants gave their consent to anonymously publish their entered data) and by the Institutional review board (IRB) of the Faculty of Medicine at Aleppo University (reference number 1535) that was compatible with the Declaration of Helsinki.

## Availability of data and materials

All data generated or analysed during this study are included in this published article.

## Funding

There are no funding sources.

## Provenance and peer review

Not commissioned, externally peer-reviewed.

## Registration of research studies

Registration of research studies, We registered our study in ResearchRegistry in accordance with the Declaration of Helsinki. Our number registry is researchregistry7939 (https://www.researchregistry.com/browse-the-registry#home/registrationdetails/628a27ad01b732001e2f81e7).

## Guarantor

Moustafa Alhashemi moustafa.alhashmy@gmail.com.

## Declaration of competing interest

The authors declare that they have no competing interests.
